# Predicting Suicide Attempts among Major Depressive Disorder Patients with Structural Neuroimaging: A Machine Learning Approach

**DOI:** 10.1192/j.eurpsy.2023.2364

**Published:** 2023-07-19

**Authors:** L. Fortaner-Uyà, C. Monopoli, F. Calesella, F. Colombo, B. Bravi, E. Maggioni, E. Tassi, S. Poletti, I. Bollettini, F. Benedetti, B. Vai

**Affiliations:** 1 University Vita-Salute San Raffaele, Division of Neuroscience; 2 IRCCS San Raffaele Hospital, Psychiatry and Clinical Psychobiology Unit - Division of Neuroscience; 3 Politecnico di Milano, Department of Electronics - Information and Bioengineering; 4 Fondazione IRCCS Ca’ Granda Ospedale Maggiore Policlinico, Department of Neurosciences and Mental Health, Milan, Italy

## Abstract

**Introduction:**

Every year at least one million people die by suicide, with major depressive disorder (MDD) being one of the major causes of suicide deaths. Current suicide risk assessments rely on subjective information, are time consuming, low predictive, and poorly reliable. Thus, finding objective biomarkers of suicidality is crucial to move clinical practice towards a precision psychiatry framework, enhancing suicide risk detection and prevention for MDD.

**Objectives:**

The present study aimed at applying machine learning (ML) algorithms on both grey matter and white-matter voxel-wise data to discriminate MDD suicide attempters (SA) from non-attempters (nSA).

**Methods:**

91 currently depressed MDD patients (24 SA, 67 nSA) underwent a structural MRI session. T1-weighted images and diffusion tensor imaging scans were respectively pre-processed using Computational Atlas Toolbox 12 (CAT12) and spatial tract-based statistics (TBSS) on FSL, to obtain both voxel-based morphometry (VBM) and fractional anisotropy (FA) measures. Three classification models were built, entering whole-brain VBM and FA maps alone into a Support Vector Machine (SVM) and combining both modalities into a Multiple Kernel Learning (MKL) algorithm. All models were trained through a 5-fold nested cross-validation with subsampling to calculate reliable estimates of balanced accuracy, specificity, sensitivity, and area under the receiver operator curve (AUC).

**Results:**

Models’ performances are summarized in Table 1.

**Table 1.** Models’ performances.

**Input features**	**Algorithm**	**Specificity**	**Sensitivity**	**Balanced accuracy**	**AUC**
VBM	SVM	55.00%	50.00%	52.50%	0.55
FA	SVM	72.00%	54.00%	63.00%	0.62
VBM and FA	MKL	68.00%	54.00%	61.00%	0.58

*Abbreviations: AUC, area under the receiver operator curve; FA, fractional anisotropy; VBM, voxel-based morphometry.*

**Conclusions:**

Overall, although overcoming the random classification accuracy (i.e., 50%), performances of all models classifying SA and nSA MDD patients were moderate, possibly due to the imbalanced numerosity of classes, with SVM on FA reaching the highest accuracy. Thus, future studies may enlarge the sample and add different features (e.g., functional neuroimaging data) to develop an objective and reliable predictive model to assess and hence prevent suicide risk among MDD patients.

**Disclosure of Interest:**

None Declared

